# The Practice Experience of an Adult Reconstruction Surgeon: A Cross-Sectional Analysis and Survey of the American Association of Hip and Knee Surgeons Membership

**DOI:** 10.1016/j.artd.2024.101328

**Published:** 2024-06-27

**Authors:** David E. DeMik, Anna Cohen-Rosenblum, David C. Landy, Joshua Kerr, Justin T. Deen, Prem N. Ramkumar, Jenna Bernstein

**Affiliations:** a Rockhill Orthopaedic Specialists, Lee’s Summit, MO, USA; b Department of Orthopaedic Surgery, Louisiana State University, New Orleans, LA, USA; c University of Kentucky College of Medicine, Lexington, KY, USA; d American Association of Hip and Knee Surgeons, Rosemont, IL, USA; e Florida Orthopaedic Institute, Ocala, FL, USA; f Long Beach Orthopaedic Institute, Long Beach, CA, USA; g Connecticut Orthopaedics, Trumbull, CT, USA

## Abstract

**Background:**

As demand for total hip arthroplasty and total knee arthroplasty increases, more surgeons have pursued subspecialty training in adult reconstruction. However, little information is available regarding the practice environment in which these fellowship-trained surgeons practice. The purpose of this study was to describe the practice environments of contemporary adult reconstruction surgeons.

**Methods:**

A survey was developed and distributed to members of the American Association of Hip and Knee Surgeons from December 2022 to January 2023. Information was collected on surgeon demographics, practice setting, call requirements, and educational debt. Responses were recorded using frequencies and proportions.

**Results:**

A total of 886 of 2471 (36%) surgeons completed the survey, with 93% identifying as male and 81% as white. The primary surgical practice locations were: community hospital 53%, academic/tertiary hospital 24%, specialty orthopedic hospital 17%, and ambulatory surgery center 7%. Nearly half (49%) of the respondents practiced in orthopedic specialty groups, and 60% spent 50%-66% of their clinical time in the office. The majority of surgeons performed between 101-250 (20%) and 251-400 (31%) arthroplasty cases per year, though this varied considerably. Call was taken by 77% of surgeons, yet only 54% received compensation.

**Conclusions:**

The most common practice setting for adult reconstruction surgeons was in a community-based hospital as part of a large orthopedic specialty group. Despite the considerable variability in annual procedure volume, the majority of surgeons spent over half their clinical time in office and had call obligations with variable compensation models.

## Introduction

The demand for total hip arthroplasty (THA) and total knee arthroplasty (TKA) is increasing as the US population continues to age. By 2030, annual THA and TKA volumes are projected to grow to 635,000 and 1.26 million procedures, respectively. [1] To meet this increased demand, many graduating orthopedic surgery residents are choosing to pursue subspecialty training in adult reconstruction. Both the number of programs and positions available have increased, with a total of 213 fellowship positions available through 112 training programs in the 2022 fellowship match process [https://www.aahks.org/the-match/]. There has also been an increase in jobs for fellowship-trained adult reconstruction surgeons, and recent fellowship graduates perform the majority of their surgical procedures within their area of subspecialty training. [2,3]

Despite the increase in both the frequency at which these procedures are performed and number of surgeons with adult reconstruction subspecialty training, little information is available regarding the breadth of circumstances under which these surgeons practice. Given the rapidly changing contemporary healthcare environment, it is important to understand current practice conditions. Poor understanding of working conditions places surgeons at a disadvantage when evaluating jobs and negotiating contracts and may contribute to the high rate of job changes reported among early-career surgeons. [4] Additionally, understanding the current state of practice for adult reconstruction surgeons allows for continued assessment of trends. The purpose of this study was to evaluate the contemporary practice environment for adult reconstruction surgeons by surveying a large national professional society.

## Material and methods

This was a cross-sectional study involving an electronic survey distributed to members of the American Association of Hip and Knee Surgeons (AAHKS). The survey included a total of 45 questions, was organized as a single section, and was developed by a committee within AAHKS. Responses to questions were assessed individually, and there was no composite scoring. There was no pretesting of the study performed given that the topics were descriptive regarding factors related to the respondent’s practice. The survey was distributed via email on December 20, 2022, with 2 additional reminder emails on January 3 and 10, 2023. The eligibility criteria included practicing surgeon “candidate” or “fellow” members of AAHKS who perform hip and knee replacements. AAHKS is the largest organization in the US of orthopedic surgeons who perform hip and knee replacements, including many who are fellowship-trained. The survey collected data on demographics, practice characteristics, call requirements, and educational debt. This study was reviewed by an institutional review board and granted a nonhuman subject research exemption. Survey data was collected anonymously.

Demographic and practice characteristics were described using frequencies and proportions. Large orthopedic practices were defined as those with 10 or more physicians, and small orthopedic practices had fewer than 10 physicians. Practice characteristics were stratified by years in practice (1-5, 6-10, 11-20, and >20 years) and practice type. No power analysis or sample size requirement testing were performed in advance, as the goal was to survey a large specialty organization regarding practice characteristics with a goal of obtaining as many responses as possible. Missing data from unanswered survey questions was not excluded, and the remainder of the completed survey was included in the final analysis. Statistical analysis was performed using STATA Version 17.0 (StataCorp, College Station, TX).

## Results

The survey was distributed to 2471 eligible AAHKS members, and 886 surgeons (36%) completed the survey. 826 respondents (93%) were male, and the majority identified as white (81%). Practice geographic location within the US was Eastern for 276 (31%), Southern for 258 (29%), Midwest for 184 (21%), and West for 166 respondents (19%). Complete demographic information is provided in Table 1.

**Table 1:** Demographic characteristics of survey respondents.

Characteristic	N (%)
Gender	
Woman	48 (5%)
Man	826 (93%)
Not reported	12 (1%)
Years in practice	
0-5	206 (23%)
6-10	184 (21%)
11-20	195 (22%)
20 or more	301 (34%)
Race/ethnicity	
White	714 (81%)
Asian	62 (7%)
Underrepresented minority	64 (7%)
Not disclosed	46 (5%)
Geographic location	
Eastern	276 (31%)
Southern	258 (29%)
Midwestern	184 (21%)
Western	166 (19%)

Table 2 contains information on practice characteristics. Nearly half of respondents worked in large (30%) and small or solo (19%) orthopedic practices. Approximately 53% of respondents operated primarily in a community hospital, and 24% were in an academic or tertiary care setting. The most common number of hours worked per week was between 51 and 60 hours (29%), and 60% reported that between 50 and 66% of their clinical time was spent performing office hours. Median proportion of time in clinic was higher for surgeons 1-5 years into practice (Table 3). The most common annual case volumes were: 251-400 (31%), 101-250 (20%), and 401-550 (16%). 64% of surgeons had 2 operative days per week, and 24% had 3 operative days. Surgeons were most often able to perform 3 (46.9%) or 4 (30.8%) arthroplasty cases per room per day. Respondents who were 1-5 years into practice reported significantly higher educational debt at the completion of training compared to those who had been in practice for longer (Table 4).

**Table 2:** Practice characteristics of survey respondents.

Characteristic	N (%)
Practice type	
Large orthopedic group (≥10)	268 (30%)
Small orthopedic group (<10) or solo	168 (19%)
Hospital-based	236 (27%)
Academic	125 (14%)
Other	89 (10%)
Primary operative setting	
Community hospital	466 (53%)
Academic or tertiary hospital	208 (24%)
Specialty hospital	150 (17%)
Ambulatory surgery center	59 (7%)
Hours worked per week	
40 or less	94 (11%)
41-50	192 (22%)
51-60	258 (29%)
61-70	137 (15%)
71 or more	69 (8%)
Not disclosed	136 (15%)
Percent clinical time in clinic	
Less than 33%	41 (6%)
33%-49%	188 (25%)
50%-66%	447 (60%)
More than 66%	64 (9%)
Call burden per month	
No call	205 (23%)
1 d	57 (6%)
2-5 d	410 (46%)
6-10 d	171 (19%)
11 or more days	43 (5%)
Volume of cases, annual	
100 or less	40 (5%)
101-250	179 (20%)
251-400	271 (31%)
401-550	143 (16%)
551-700	59 (7%)
701 or more	45 (5%)
Not disclosed	149 (17%)

**Table 3:** Median percent clinic time by practice type and years in practice.

Practice type	Years in practice			
	1-5	6-10	11-20	Over 20
Large orthopedic group	56% (50-61)	50% (42-59)	50% (44-56)	50% (44-60)
Small orthopedic group or solo	58% (50-63)	50% (47-57)	51% (42-60)	50% (44-61)
Hospital-based	56% (50-60)	50% (42-56)	50% (50-60)	50% (50-69)
Academic	50% (43-50)	50% (38-50)	43% (38-50)	50% (44-64)

**Table 4:** Loan characteristics by years in practice.

	Years in practice			
Characteristic	1-5 N (%)	6-10 N (%)	11-20 N (%)	Over 20 N (%)
Amount after training (1000’s $)				
Less than 100	62 (36%)	58 (37%)	70 (43%)	195 (77%)
100-199	21 (12%)	38 (25%)	60 (37%)	32 (13%)
200-299	37 (21%)	24 (15%)	24 (15%)	16 (6%)
300 or more	54 (31%)	35 (23%)	10 (6%)	9 (4%)

The majority of surgeons reported taking orthopedic call, with 2-5 days per month being the most frequent (46%) (Table 5). The median number of call days per month was 3.5 for surgeons aged 1-5, 6-10, and 11-20 years in practice and across all practice types. For those in practice for >20 years, physicians in small orthopedic groups had a higher median number of call days (Table 6). Only 54% of respondents reported being paid for providing on-call coverage, while 46% were not provided additional compensation. Arthroplasty-specific call obligations were reported by 20% of respondents, and 97% did not receive any additional compensation.

**Table 5:** Call characteristics of respondents who take call.

Characteristic	N (%)
Call paid	
Yes	366 (54%)
No	315 (46%)
Arthroplasty-specific call	
Yes	133 (20%)
No	548 (80%)
Arthroplasty-specific call paid	
Yes	4 (3%)
No	129 (97%)

**Table 6:** Median number of calls per month by practice type and years in practice.

Practice type	Years in practice			
	1-5	6-10	11-20	Over 20
Large orthopedic group	3.5 (3.5-3.5)	3.5 (3.5-3.5)	3.5 (3.5-3.5)	1 (0-3.5)
Small orthopedic group or solo	3.5 (3.5-8)	3.5 (1-8)	3.5 (0-3.5)	3.5 (0-8)
Hospital-based	3.5 (3.5-8)	3.5 (3.5-8)	3.5 (3.5-8)	1 (0-3.5)
Academic	3.5 (1-3.5)	3.5 (3.5-3.5)	3.5 (3.5-3.5)	1 (0-3.5)

## Discussion

This survey found considerable heterogeneity among the practice experiences of surgeons specializing in adult reconstruction. Overall, it was most common for adult reconstruction surgeons to operate in a community-based hospital setting and as part of a small or large orthopedic specialty group. Respondents most commonly reported working an average of 51-60 hours per week. However, there was substantial variation in the number of hours worked, with 11% and 8% of respondents reporting working less than 40 or greater than 71 hours per week, respectively.

The majority of respondents performed between 101 and 400 arthroplasty cases per year. However, there was a considerable range in annual volume, with some performing less than 50 cases per year and others performing over 800. This may reflect a combination of institutional support to perform a high volume of arthroplasty procedures or flexibility within certain practices to adjust clinical volume based on surgeon preference. It was not directly assessed whether surgeons routinely had access to a “swing room” or were often “running two rooms” with overlapping procedures. Both of these practices have previously been demonstrated as safe and may be associated with higher surgical volumes. [5,6] The proportion of clinic time was slightly higher for surgeons earlier in practice. This was likely multifactorial, related to both relatively limited OR availability, as is common in many practices, and the need for additional clinic time as early-career surgeons grow their practice. Interestingly, time in clinic did not decrease substantially as the number of years in practice increased, and the median time in clinic remained around 50%. In contrast, a national survey of orthopedic trauma surgeons found 66% of respondents performed between 1 and 1.5 days of office hours per week. [7] This suggests that surgeons may need to dedicate approximately half of their clinical time to office hours in order to maintain a steady, elective surgical practice, regardless of years of experience. This also highlights the substantial effort required in the preoperative and postoperative periods to educate patients regarding surgical and perioperative expectations and, in general, provide high-value arthroplasty care. [8,9] Payers should be aware of these efforts when determining reimbursement rates for global periods of care.

The majority of arthroplasty surgeons in this study reported having a requirement to take general orthopedic calls, with arthroplasty-specific call coverage being less frequent. Just over half of respondents reported receiving direct compensation for providing call coverage. These findings were similar to a national survey of orthopedic trauma surgeons, in which 54% reported direct compensation for call duties. [7] The median number of call days per month was 3.5, and this did not decrease until surgeons were >20 years into practice. Less than half of respondents took an arthroplasty-specific call, and it was rarely reimbursed. As more THA and TKA procedures are performed on younger patients, there will be an increase in arthroplasty-specific complications, such as periprosthetic fractures and periprosthetic joint infections. These patients often require acute care from surgeons with expertise in adult reconstruction, which may lead to an increased need for a subspecialty call. As this occurs, surgeons must be provided with adequate resources and reimbursement to treat patients with these complex problems.

The findings also highlight a discordance between the practice environment for surgeons performing adult reconstructive surgery and the fellowship training environments. Many fellowship programs are based in large academic centers or highly developed specialty practices; however, the majority of respondents practiced in smaller, community-based settings. While there is clear value in how these training environments provide exposure to high-complexity adult reconstructive problems and a large case volume, it is possible that some aspects of this training may not be completely translatable to the practice environment of many surgeons. There may be value in hybrid-type programs where trainees are both exposed to problems at a tertiary center and also at the community level with some inclusion of general orthopedics.

Surgeons who were earlier in their careers reported overall higher amounts of educational debt at the completion of training. This is likely related to the continually rising costs of undergraduate and medical education, but may also be related to increasing lengths of training as more trainees pursue gap years, research years, and additional fellowship training. The rising debt levels of surgeons entering practice are particularly noteworthy when considering recent reductions in the relative value units and associated physician compensation for THA and TKA procedures. Higher anticipated debt levels coupled with reductions in reimbursement may dissuade otherwise qualified surgeons-in-training from pursuing subspecialty training in adult reconstruction.

This study is not without limitations. The survey was only distributed to members of AAHKS, and not all surgeons in the US who perform THA and TKA are AAHKS members. Additionally, not all AAHKS members responded to the survey, and the findings may be influenced by response bias. However, the respondents reported practicing in a variety of employment models, geographic locations, and at various points throughout their career, and this seems to be an appropriate sample of adult reconstruction surgeons practicing in the US. This was a cross-sectional study and therefore does not assess changes in practice that may have occurred over time. Additionally, this study did not directly assess burnout or job satisfaction as it relates to practice environment, surgical volume, or hours worked. This is an important area that warrants further study.

## Conclusions

This study presents novel, national survey data to better quantify the practice environment for contemporary arthroplasty surgeons. There was considerable variability in practice location, employment structure, and annual procedural volume. Despite this heterogeneity, the majority of respondents dedicated approximately half of their clinic time to the office setting and had a requirement to take general orthopedic call throughout their career. Data from this survey is an important reference for evaluating future changes in the practice environment and has identified a number of areas for future investigation, including emergence of arthroplasty-specific call responsibilities, the impact of growing revision burden, the factors contributing to the wide range of case volume, and the influence of educational debt on surgeon subspecialty selection.

## Conflicts of interest

P. Ramkumar receives royalties from Globus and is a paid consultant for Stryker and Globus. He is an unpaid consultant for Overture Resurfacing, Best in Class MD; receives stock options from Intelligent Health Analytics Inc. and Johnson & Johnson; and is an editorial/governing board member of the Journal of Arthroplasty. J. Kerr has stock options in Smith and Nephew. D. DeMik is an editorial/governing board member of the Journal of Arthroplasty. J. Bernstein is a paid consultant for Depuy Synthes and Smith & Nephew and is a board member of AAHKS, AAOS, EOA, and CT Ortho Society. A. Cohen-Rosenblum is a speaker bureau of Microport; receives royalties/financial/material support from Elseiver and JBJS; is an editorial board member of Arthroplasty Today and Journal of Arthroplasty; and is a board/committee member of AAOS, RJOS, and AAHKS. J. Deen is a paid consultant for OrthoDevelopment Corporation; receives research support from Depuy Synthes; is a peer reviewer of Journal of Arthroplasty; and is a member of the Board of Directors of Florida Orthopedic Society serving as Vice Chair and Quality Measures Committee of the American Association of Hip and Knee Surgeons. D. Landy is a speaker bureau member of Smith and Nephew; is an editorial/governing board member of Am J Sports Med; and is a board member of AAHKS and YAG.

For full disclosure statements refer to https://doi.org/10.1016/j.artd.2024.101328.

## CRediT authorship contribution statement

**David E. DeMik:** Writing – original draft. **Anna Cohen-Rosenblum:** Conceptualization, Writing – review & editing. **David C. Landy:** Data curation, Formal analysis. **Joshua Kerr:** Methodology, Project administration, Resources. **Justin T. Deen:** Conceptualization, Writing – review & editing. **Prem N. Ramkumar:** Writing – review & editing. **Jenna Bernstein:** Conceptualization, Writing – review & editing.

## Appendix A Supplementary data

Conflict of Interest Statement for Cohen-Rosenblum

Conflict of Interest Statement for Bernstein

Conflict of Interest Statement for DeMik

Conflict of Interest Statement for Deen

Conflict of Interest Statement for Kerr

Conflict of Interest Statement for Landy

Conflict of Interest Statement for Ramkumar
