# Supplementary material for: The Practice Experience of an Adult Reconstruction Surgeon: A Cross-Sectional Analysis and Survey of the American Association of Hip and Knee Surgeons Membership
Source: Arthroplast Today. 2024 Jun 27;27:101328. doi: 10.1016/j.artd.2024.101328 (PMC11282424; doi:10.1016/j.artd.2024.101328)
Supplement: Conflict of Interest Statement for Cohen-Rosenblum [file mmc1.pdf]

CONFLICT OF INTEREST STATEMENT

American Association of Hip and Knee Surgeons

(Adopted from the American Academy of Orthopaedic Surgeons disclosure statement)

The following form **must be filled out completely and submitted by each author (example, 6 authors, 6 forms). All items require a response. If there is no relevant disclosure for a given item, enter "*None*."**

Manuscript Title

1. Royalties from a company or supplier (The following conflicts were disclosed)

none

2. Speakers bureau/paid presentations for a company or supplier (The following conflicts were disclosed)

microport

3A. Paid employee for a company or supplier (The following conflicts were disclosed)

none

3B. Paid consultant for a company or supplier (The following conflicts were disclosed)

none

3C. Unpaid consultants for a company or supplier (The following conflicts were disclosed)

none

4. Stock or stock options in a company or supplier (The following conflicts were disclosed)

none

5. Research support from a company or supplier as a Principal Investigator (The following conflicts were disclosed)

none

6. Other financial or material support from a company or supplier (The following conflicts were disclosed)

none

7. Royalties, financial or material support from publishers (The following conflicts were disclosed)

Elseiver
JBJS

8. Medical/Orthopaedic publications editorial/governing board (The following conflicts were disclosed)

Arthroplasty Today
Journal of Arthroplasty

9. Board member/committee appointments for a society (The following conflicts were disclosed)

AAOS, RJOS, AAHKS

Each author must sign AND print or type his/her name, date and submit a separate form

In addition, one BLINDED Conflict of Interest form (no author names used) should be submitted per manuscript with all author disclosures.

Anna Cohen-Rosenblum	[signature]	9/7/23
Author Name (Print or Type)	Author Signature	Date
